# The MEDEA FAR-EAST Study: Conceptual framework, methods and first findings of a multicenter cross-sectional observational study

**DOI:** 10.1186/s12873-019-0240-7

**Published:** 2019-05-02

**Authors:** Sophia Hoschar, Jiangqi Pan, Zhen Wang, Xiaoyan Fang, Xian’e Tang, Weiqi Shi, Rongxiang Tu, Peng Xi, Wenliang Che, Hongbao Wang, Yawei Li, Kurt Fritzsche, Xuebo Liu, Karl-Heinz Ladwig, Wenlin Ma

**Affiliations:** 1 Institute of Epidemiology II, Mental Health Research Unit, Helmholtz Zentrum München, German Research Center for Environmental Health (GmbH), Ingolstädter Landstr 1, 85764 Neuherberg, Germany; 2 Department of Cardiology, Tongji-Hospital, Tongji-University, Shanghai, People’s Republic of China; 3 Department of Cardiology, Tenth-Hospital, Tongji-University, Shanghai, People’s Republic of China; 4 Department of Cardiology, Yangpu-Hospital, Tongji-University, Shanghai, People’s Republic of China; 5 Department of Cardiology, 455-Hospital, Tongji-University, Shanghai, People’s Republic of China; 6 Department of Psychosomatic Medicine and Psychotherapy, Medical Center- University of Freiburg, Faculty of Medicine, Freiburg, Germany; 7 Department of Psychosomatic Medicine and Psychotherapy, Technical University Munich, Munich, Germany

**Keywords:** Acute myocardial infarction, Prehospital delay, Cross-sectional observational, Multicenter study, China

## Abstract

**Background:**

The substantial increase in cardiovascular diseases (CVD) in China over the last three decades warrants comprehensive preventive primary and secondary strategies. Prolonged prehospital delay (PHD) has been identified as a substantial barrier to timely therapeutic interventions for acute myocardial infarction (AMI). Despite worldwide efforts to decrease the patient’s decision-making time, minimal change has been achieved so far. Here, we aim to describe the conceptual framework and methods and outline key data of the MEDEA FAR-EAST Study, which aimed to elucidate in-depth barriers contributing to delay in Chinese AMI-patients.

**Methods:**

Data sources of this multicenter cross-sectional observational study are a standardized bedside interview, a self-administered tailored questionnaire tool and the patient chart. PHD was defined as the main outcome and triangulated at bedside. Standard operation procedures ensured uniform data collection by trained study personnel. The study was ethically approved by Tongji-Hospital and applied to all participating hospitals.

**Results:**

Among 379 consecutively screened patients, 296 (78.1%) fulfilled eligibility criteria. A total of 241 (81.4%) AMI-patients were male and 55 (18.6%) female. Mean age was 62.9 years. Prehospital delay time was assessed for 294 (99.3%) patients. Overall median PHD was 151 min with no significant sex difference. Symptom mismatch was present in 200 (69.7%) patients and 106 (39.0%) patients did not attribute their symptoms to cardiac origin. A total of 33 (12.4%) patients suffered from depression, 31 (11.7%) from anxiety and 141 (53.2%) patients employed denial as their major coping style.

**Conclusion:**

This is the first study on prehospital delay with emphasis on psychological variables in Chinese AMI-patients. A comprehensive assessment tool to measure clinical and psychological factors was successfully implemented. Socio-demographic key data proved a good fit into preexisting Chinese literature. Potential barriers including cardiac denial and symptom-mismatch were assessed for the first time in Chinese AMI-patients. The pretested selection of instruments allows future in depth investigations into barriers to delay of Chinese AMI-patients and enables inter-cultural comparisons.

## Background

In China, the burden of cardiovascular disease (CVD) is substantially increasing and is now the leading cause of death [1], accounting for a 1-year incidence of two million acute myocardial infarction (AMI) patients in China in the year 2011 [2]. AMI was responsible for 64.25 deaths per 100.000 inhabitants in 2014 [3].

Therapeutic interventions of AMI are highly time-dependent [4]. Despite recommendations by international guidelines to arrive at the hospital door within 2 h upon symptom-onset, prehospital delay (PHD) remains a global obstacle to timely treatment [4, 5]. A recent worldwide review on prehospital delay which included studies from Southeast Asia and China has estimated median prehospital delay time to range between 1.6-12.9 h [6]. The major component of PHD is patient-related delay [7], which is widely acknowledged to account for about 75% of overall prehospital delay [8]. Up to now, a total of eight internationally recognized clinical studies on delay time during AMI have been performed in China [9–16] (see Table 1). Compared to estimates of median delay times in high-income countries [17], median prehospital delay times in these Chinese investigations ranged in a relatively favorable time window of 130 to 150 min [9, 10, 12, 13, 15], nevertheless showing that still more than half of Chinese MI-patients fail to reach emergency facilities within the recommended time window of 120 min.

**Table 1:** Chinese prehospital delay studies published in English language (2016–2004)

Reference	Zhang B et al. 2016 [15]	Peny YG et al. 2014 [10]	Wang X and Hsu L 2013 [13]	Gao Y and Zhang HJ 2013 [9]	Qian L et al. 2013 [11]	Song L et al. 2010 [12]	Zhang S et al. 2009 [16]	Wu Y et al. 2004 [14]
Title	Gender and Age Differences Associated with Prehospital Delay in Chinese Patients presenting with ST-Elevation Myocardial Infarction	Factors associated with prehospital delay in patients with ST-segment elevation acute myocardial infarction	Treatment-seeking delays in patients with acute myocardial infarction and use of the emergency medical service	The effect of symptoms on prehospital delay time in patients with acute myocardial infarction	Factors associated with decision time for patients with ST-segment elevation acute myocardial infarction	Impact of patients’ symptom interpretation on care-seeking behaviors of patients with acute myocardial infarction	Use of Emergency Medical Services in Patients with Acute Myocardial Infarction in China	Factors associated with the extent of care-seeking delay for patients with acute myocardial infarction in Beijing
Setting/ No. of hospitals	Liaoning Province/ 20	Beijing/ 1	Shanghai/ 3	Jinzhou/ 1	Whenzhou/ 1	Beijing/ 19	Beijing/ 21	Beijing/ 8
Study	prospective, cross-sectional, multi-center	cross-sectional	cross-sectional, multi-center	cross-sectional	cross-sectional	cross-sectional, multi-center	prospective, cross-sectional, multi-center	cross-sectional, multi-center
Sample size, diagnosis	*n* = 1429, STEMI	*n* = 1088, STEMI	*n* = 250, AMI	*n* = 116, AMI	*n* = 100, AMI	*n* = 799, STEMI	*n* = 803, AMI	*n* = 102. AMI
Reported results								
Sex	*27.2% female*	*24.9% female*	*36.4% female*	*27.6% female*	*26% female*	*21.1% female*	*22.0% female*	*23.5% female*
Mean age (+/− SD)	*–*	*60.94 yrs (+/−12.8)*	*63.5 yrs*	*64.1 yrs (+/− 13.9)*	*65.7 yrs (+/− 12.7)*	*61 yrs (+/− 13)*	*–*	*62 yrs (+/− 12)*
Years of education	65.7% (> 6 yrs)	47.2% (> 9 yrs)	*78.0% (> 6 yrs)*	–	38% (> 6 yrs)	16.3% (≥12 yrs)	57.2% (> 6 yrs)	75.5% (> 9 yrs)
Insurance	*58.1%*	*–*	*88.8%*	*–*	*89.0%*	*76.2%*	*73.7%*	*–*
Reinfarction	*7.3%*	*24%*	*10.8%*	*33.6%*	*–*	*9.5%*	*9.5%*	*11.8%*
Chest pain	*73.8%*	*84.7%*	*–*	*68.1%*	*–*	*82.1%*	*87.6%*	*87.3%*
Angina	28.4%	*–*	*–*	*–*	*24.0%*	*–*	*25.9%*	*33.3%*
EMS-use	*–*	*59%*	*30.8%*	*–*	*–*	*33.0%*	*39.5%*	*14.7%*
Median delay time	150 min	130 min	130min* Median patient decision time	132 min	–	140 min	–	–
Delay > 2 h	53.7%	51.4%	–	66.7%	–	55.4%	–	63%

* = patient decision time

Substantial efforts have been undertaken to reduce patient-related prehospital delay in numerous populations and secondary based prevention campaigns [18], yet, the results were mostly disappointing [19]. Recently, positive effects on prehospital delay could be demonstrated in interventions that specifically addressed subjective risk-perception in patient decision-making [20, 21], providing a promising concept for future preventive efforts. Unfortunately, the major bulk of delay research in both the western world and in China (see Table 1) is currently restricted to data assessments on sociodemographic and symptom-related variables which do not target those “psychological barriers” increasingly acknowledged as major drivers [22] in the final decision to dispatch emergency medicine services (EMS) [23].

At AMI-onset, several factors such as slow symptom-onset, intermittent symptoms, symptom vagueness [24] and experiencing symptoms which do not match one’s expectations of AMI-symptoms [25] or are attributed to causes other than the heart can act as barriers to recognizing symptoms in a timely manner. Additionally, the decision process to seek immediate medical help may be compromised by low perceived personal risk or by health attitudes like cardiac denial [26] even when symptoms are acknowledged as cardiac in origin [27].

Little is known about the impact of depression, anxiety and other psychosocial conditions on the delay process. It is yet not unlikely that depressed patients might delay due to a lack of motivation and energy to seek help [28] whereas anxiety may sharpen the patients self-perceived risk and thus render them more capable of making decisions to get immediate help [29]. Though generalized anxiety may decrease delay time [29], insufficient knowledge of the time dependent nature of treatment as well as being afraid of causing a “false alarm” or “not wanting to bother the physicians” might keep patients from seeking professional help [30].

The MEDEA Study in Munich (Germany, 2007–2012) was developed to overcome shortcomings in delay research focusing on symptom patterns only, and to broaden the horizons of this research. It provided a conceptual framework and subsequent data assessment to identify barriers to help-seeking behavior during an acute infarction situation. The multicenter cross-sectional MEDEA FAR-EAST Study was designed to replicate this study, with its major objective being the application of a holistic approach in addressing patient-related delay by examining somatic, clinical, health-psychology related concepts and influential affective factors on prehospital delay in Chinese AMI-patients. The preselection of instruments was guided by (a) theoretical framework, (b) validated instruments in the Chinese language and (c) and the applicability within the particular Chinese cultural background. The aim of this paper is to present the framework, methods and descriptive study data on prehospital delay times as well as factors related to patient characteristics and symptom-onset.

## Methods

### Study design

The main inclusion criterion was hospitalization with an acute myocardial infarction (AMI), confirmed by typical symptoms at onset, and elevated cardiac biomarkers (troponin I or troponin T) as well as corresponding ECG-diagnosis. No restrictions were made regarding age and sex. Exclusion criteria were an out-of-hospital cardiac arrest as well as cognitive impairment and language barrier.

### Study setting

Shanghai is the largest city in China with a total population of 24.2 million people and an average population density of 3816 people per km^2^ in its urban areas [31]. Across the nation, Shanghai has the largest percentage of aging population (> 65 years) accounting for 18.1% compared to the national average of 10.1% [32]. Furthermore, life expectancy among Shanghai residents is among the highest in China with a mean age of 83.2 years in 2016 [31]. Combined with decreasing birth rates, this confronts Shanghai with an overall aging population. In China, 90% of inhabitants have basic health insurance [30]. This health insurance, however, varies in its coverage depending on population groups and registration area, and patients have to pay variable shares. Family physicians present only a negligible 5.6% of all Chinese physicians [﻿33﻿]. Instead, the majority of doctor visits (90% in 2012) take place in outpatient departments of public hospitals which results in overcrowding, long waiting times and short patient-doctor encounters [34]. Medical insurance covers all expenses for emergency treatments in public hospitals, nevertheless, non-life-threatening emergency-department visits and hospitalizations require the patient to pay a deposit [35]. Ambulance services are only used among a third of AMI-patients [13], possibly due to costs, low efficacy or missing awareness of emergency medical services. Insurance covers a variable proportion of the fee that is charged for ambulance use [36].

#### Clinical setting and organization

The patient sample was recruited from four cardiology departments in Shanghai, all providing acute coronary care units. After preselection of instruments (by S. Hoschar et al.), a Chinese version of the study protocol was presented to the Ethics Commission of the Tongji-University affiliated Tongji-Hospital (by W. Ma). The Commission approved the study on 16th of March 2016 (伦审-KYSB-2016-74). The ethics approval applied to all participating centers. Recruitment started in Tongji-Hospital in Mid-April together with Yangpu-Hospital and 455 People’s Hospital. By end of July, recruitment was expanded to Tenth-Hospital. Recruitment was ended in Mid-January 2017.

Patient inclusion and data collection were performed within two days after hospitalization (SD: +/− 1.87 days, *n* = 86). On average, between one to 2 h were spent by trained personnel conducting the interview and assisting patients with completing the questionnaire. Regular newsletters informed all study-affiliated members of the study’s progress and developments. The study personnel received training prior to their participation and were closely monitored during the data acquisition process in order to avoid reporting bias. Standard operating procedures (SOP) were implemented to ensure the training of new members and these methods were kept consistent for all participating hospitals.

### Data sources

The data collection procedure was divided into three parts. First, a bedside interview was conducted with trained study personnel. After this, a self-administered questionnaire was handed to the patient for self-assessment. Lastly, basic epidemiological and medical information were collected from the hospitals’ patient charts. The patients were followed up at bedside or via telephone calls for any missing or incoherent answers.

#### Information from the interview

##### Assessment of prehospital delay times

Prehospital delay time was the primary outcome defined as the time interval between symptom-onset and arrival at the hospital door, measured in minutes. Symptom-onset was clearly defined as symptoms that worsened or stayed continuous without decreasing over time. Nevertheless, defining symptom-onset remained a challenge (for example, patients often had difficulties to differentiate between prodromal symptoms and intermittent acute onset). The onset-time was triangulated by trained personnel in the interview, using events from the patient’s daily routine to help them establish the chronology of symptom-onset. Patients were asked to give a broad estimate of the time of symptom-onset, which was then further specified by placing it in relation to times of regular activities, meals, sleeping habits and other routines. This technique has previously been developed and tested by Moser et al., who found that this technique enabled patients who did not initially remember onset-time to successfully recall it [37]. The hospital registration receipt (挂 号 单, Guàhàodān) was used as the hospital arrival time, either directly or through the hospital information center. Additionally, an effort was made to distinguish decision from transportation, by coding time of decision to seek help as a secondary outcome. Prompting the time of decision to seek help provided patients with an additional anchor item and followed the recommendations of Mackay et al. to collect at least two components of prehospital delay [38].

##### Health related behavior

At bedside, comprehensive data on sociodemographic and health-related behaviors were assessed (physical activity, burden of work, smoking). Health attitudes and frequency of doctor consultation prior to AMI helped to portray patient’s overall approach to health. Angina pectoris six months prior to AMI was assessed following the Rose Angina Questionnaire [39] which allowed the evaluation of any prodromal chest pain (PCP), chest pain of unknown origin (unexplained PCP), possible angina or definite angina.

##### Symptom presentation at AMI-onset

The acute onset of MI was a central part of the interview to outline details of symptom presentation. The duration, intensity and character (e.g. intermittent, increasing over time) of chest pain and/or other cardiac symptoms were documented. Patient interpretation of the onset-symptoms was measured by the items patient expectation and symptom attribution.

##### Behavioral responses to AMI and context variables

The Response of Symptoms Questionnaire [40] was used to obtain information factors contributing to delay in the following domains: (1) the context in which AMI-symptoms appeared (at home, during work etc.); (2) to address with whom the patient was and what they were doing when the signs and symptoms occurred; (3) the responses of witnesses to the patient’s symptoms; (4) the behavioral responses to symptoms (e.g. wait and see; trying to relax; calling the emergency system); (5) the affective response to the symptoms. Subjective rating of helplessness, fear of death and fear before seeking help was assessed in single-item instruments. Mode of transportation was coded in the interview as self-transportation, transportation by others and transportation via ambulance.

#### Information from the self-administered questionnaire

The self-administered questionnaire assessed variable psychometric variables including psychological characteristics, personality concepts as well as AMI-related knowledge. Except for the instrument measuring AMI-related knowledge, all psychometric instruments used were standardized instruments, summarized in detail in Table 2.

**Table 2:** Psychometric instruments of the MEDEA FAR-EAST study

	Affective disorders		Stress and Somatic symptom burden			Social support
Instrument	Major Depression Inventory	General Anxiety Disorder Scale	INTER-HEART Stress	High Symptom Somatic Scale	Perceived Stress Scale	Social Support Rating Scale
Abbreviation	MDI	GAD-7	IHS	SSS-8	PSS-4	SSRS
Authors, year	Bech P., Rasmussen N.A., Noerholm V., Abildgaard W. (2001) [41]	Spitzer R. L., Kroenke K., Williams J.B. (2006) [42]	Rosengren A. et al. (2004) [43]	Narrow W. et al. (2013) Gierck B. et al. (2014) [44]	Cohen S., Kamarck T Mermelstein R. (1983) [45]	Shuiyuan X. (1994) [46]
Measures	Depressed mood severity, major depressive disorder according to DSM-IV and ICD-10	Anxiety	Stress from the workplace, at home and financial problems	Somatic Symptom Burden	Global measure of perceived stress	Social support in Chinese patients
Domains	Measures 10 (3 core; 7 accompanying) symptoms of depression	1 domain	3 domains: stress in the family, at work or due to financial problems	Somatic, anxiety and depressive symptoms	2 domains	3 domains: subjective support, objective support and support usage
Items (n)	10	7	3	8	4	10
Range/score	0–50	0–21	3-12	0–32	0–16	12–66
Reference describing the use on a Chinese population	Sociodemographic Correlates of Unipolar Major Depression among the Chinese Elderly in Klang Valley, Malaysia: An Epidemiological Study Verma et al. (2014) [47]	Reliability and validity of a generalized anxiety disorder scale in general hospital outpatients He X. et al. (2010)* [48]	Marital Status, Education, and Risk of Acute Myocardial Infarction in Mainland China: The INTER-HEART Study Hu et al. (2012) [49]	Application of Patient Health Questionnaire somatic symptom scale in the General Hospital Outpatients Qian J. et al. (2014)* [50]	Three versions of Perceived Stress Scale: validation in a sample of Chinese cardiac patients who smoke Leung et al. (2010) [51]	The Theoretical Basis and Research and Application of “Social Support Rating Scale” Shuiyuan X. et al. (1994)* [46]
	Locus of control	Well-being	Cardiac denial	Resilience	Personality disorders	
Instrument	Mental Health locus of control scale	Well-Being Index	The cardiac denial of impact scale	Resilience Scale 5	Type D Scale-14	Framingham Type A personality scale
Abbreviation	MHLC	WHO-5	CDIS	RS-5	DS-14	FRAS
Authors, year	Wallston K.A., Wallston B.S., DeVellis R. (1978) [52]	Bech P., Olsen L.R., Kjoller M., Rasmussen N.K. (2003) [53]	Hackett T.P. and Cassem N.A.(1974) [54] Fowers B.J.(1992) [55]	von Eis enhart Rothe A. et al. (2013) [56, 57]	Denollet (2005) [58]	Haynes et al. (1980) [59]
Measures	Degree to which health is considered to be determined by own behaviour	Well-being according to the WHO definition	Impact of denial among cardiac patients	Psychosocial stress resistance	Negative affectivity, social inhibition, type D personality	Traits, qualities in character and reaction to work or housework
Domains	Self-responsibility, self-blame, powerful others	Happiness, energy, motivation, interest in daily life.	Denial of impact	Personal competence and acceptance of self and life	Negative affectivity and social inhibition	2 domains
Items (n)	12	5	8	5	14 (7 per domain)	10
Range/score	1–5 (per item)	0–100	8–40	5-35	0–28 (per domain)	0–1
Reference describing the use on a Chinese population	Clinical application study on multidimensional health locus of control scales Chen S.J., Wang W.L., Pan Q. (2014)^*^ [60]	Reliability and validity of the World Health Organization Five-item Well-being Index for detecting depressive disorders in senior middle school students Wang Z., Bian Q. (2011)* [61]	–	–	Development of type D personality scale and its reliability and validity in Chinese adolescents and children with emotion disorders. Zhang Y. et al. (2006)* [62]	Short communication: Personal and organizational outcomes related to job stress and Type-A behavior: a study of Canadian and Chinese employees Jamal M. (2005) [63]

* = published in Chinese language

We used two scales to measure affective disorders. Depression was measured using the Major Depression Inventory (MDI), a 10-domain instrument able to generate an ICD-10 diagnosis. Following the suggestion of Bech et al. [64], a cut-off point (> 25) was chosen when using the MDI as a rating scale. Anxiety was measured using a validated translation of the Generalized Anxiety Disorder Scale (GAD-7) [﻿42﻿], where scores of above or equal to 10 highlights anxious patients.

Denial regarding cardiac illness was measured in the 8-item Cardiac Denial of Impact Score (CDIS) [55]. A score of ≥25 indicates cardiac denial.

Stress was measured by two items, a) the INTER-HEART Stress Scale (IHS) [43], a 3-item instrument measuring stress in financial, family and work-related context. Furthermore, the short version of the perceived stress scale [45] depicts a global measure of stress in 4 items. The somatic symptom burden was captured by the somatic symptom scale (SSS-8) [44] which comprises 8 items to detect somatic, anxiety and depression-related symptoms.

Health locus of control was measured using the Multidimensional Health Locus of Control Scale (MHLC). It consists of 12 items and was initially developed by Wallston et al. [52] and features 3 domains.

The social support was measured by the Chinese 10-item Social Support Rating Scale (SSRS) [46]. It measures social support in 3 domains, namely subjective, objective and usage of social support.

Well-being was measured by the WHO-5 [53], a 5-item instrument developed by the WHO to measure happiness, energy, motivation and interest in daily life.

Type-D personality was measured using the Type-D Scale 14 (DS-14) which is divided into two subscales with 7 items each, measuring social inhibition and negative affectivity [58]. Type-A personality was measured using the Framingham Type A behavior score (FRAS), consisting of 10 items to measure behavior indicative of Type A [59].

Resilience was measured in a short 5-item version (RS-5) developed from the original version of the RS-14 [65]. This tool measures the domains of personal competence and acceptance of self and life.

Knowledge of AMI-symptoms and appropriate actions to be taken when experiencing those symptoms was measured in the questionnaire in two subscales. In the interview, sources of AMI-knowledge were identified, including physician, media, heart association and friends/acquaintances as possible sources.

As can be further seen in Table 2, the majority of psychometric instruments applied in the present investigation had been validated and applied in Chinese language and were published in Chinese research papers prior to our investigation [46–51, 60–63]. However, the Major Depression Inventory (MDI) [41], the INTER-HEART Stress Scale (IHS-Scale) [43], the short version of the Resilience Scale (RS-4) [57] and the Cardiac Denial of Impact Scale (CDIS) [55] were not available in Chinese. Therefore, these instruments were translated following the recommended translation guidelines of back-and-forth translation by the WHO [66]. Previous to enrollment, these four instruments were pretested in 20 patients to ensure that patients were able to understand all items.

#### Information from the hospital chart

Detail of the infarction diagnosis, elevation of cardiac biomarkers and the number of days spent in CCU, complications throughout the stay as well as therapy of the MI were documented in the hospital chart. The patient chart additionally examined if the patient was previously transferred from a hospital not capable of treating the patient for AMI.

### Data entry

The study coordinator entered the data in an Excel sheet. Checklists were created in order to monitor progress of data collection.

### Sample size calculation

It was estimated that 30% of participants would reach the hospital within < 120 min. With the assumption of a 5% error we estimated a sample size of 310 patients to ensure a power of 0.80 in proving a clinically relevant odds ratio of 2.0.

### Data analysis

Differences between continuous variables were assessed using Mann Whitney Test. All statistical analysis was run in IBM SPSS Statistics version 20 (SPSS Inc., Chicago). The distribution of delay was available as a continuous variable in minutes and heavily left-skewed, thus reporting medians delay times. Dichotomized measures of symptom expectation (≤3 vs > 3 on 5 point Likert scale) and symptom attribution (cardiac vs. non-cardiac) were used. The significance level α was set at .05. Differences in median PHD were assessed using the non-parametric Wilcoxon test. The analysis and description in this paper follow the STROBE guidelines for cross-sectional studies [67].

## Results

### Sample size and dropout-analysis

**Fig. 1:**
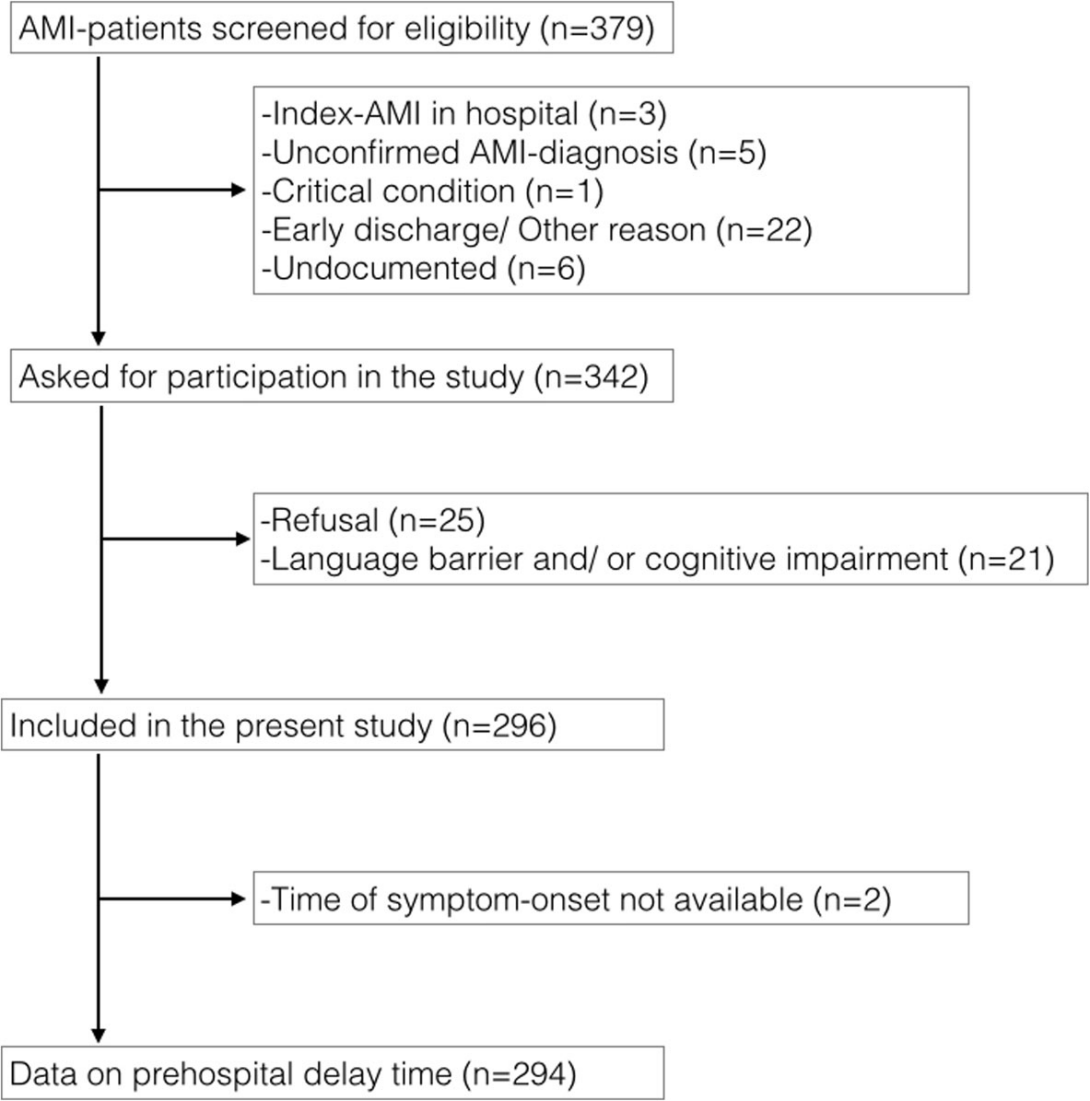
Flow-diagram for inclusion and exclusion

A total of 379 patients were considered eligible from mid-April 2016 to mid-January 2017, of which 83 (21.9%) were excluded (see Fig. 1). Causes for exclusion were refusal (*n* = 25, 30.1%) language barrier or/and cognitive impairment (*n* = 21, 25.3%), as well as other reasons such as unconfirmed diagnosis (*n* = 5, 6.0%) or missing data (*n* = 22, 26.5%). One patient (1.2%) was in a too critical condition to be interviewed and three patients (3.6%) were already in hospital when they experienced AMI. For six patients (7.2%), the reason for exclusion remained undocumented. In the present analysis, two patients had missing values of onset time, so that prehospital delay could not be determined. A dropout-analysis showed that older age was a significant factor of dropout, with patients dropping out being a mean of 69.3 years old (compared to a mean of 62.9 years in our study, *p* < 0.001). Comparison of included and excluded patients showed no significant differences in gender (*p* = 0.46).

### Patient characteristics

**Fig. 2:**
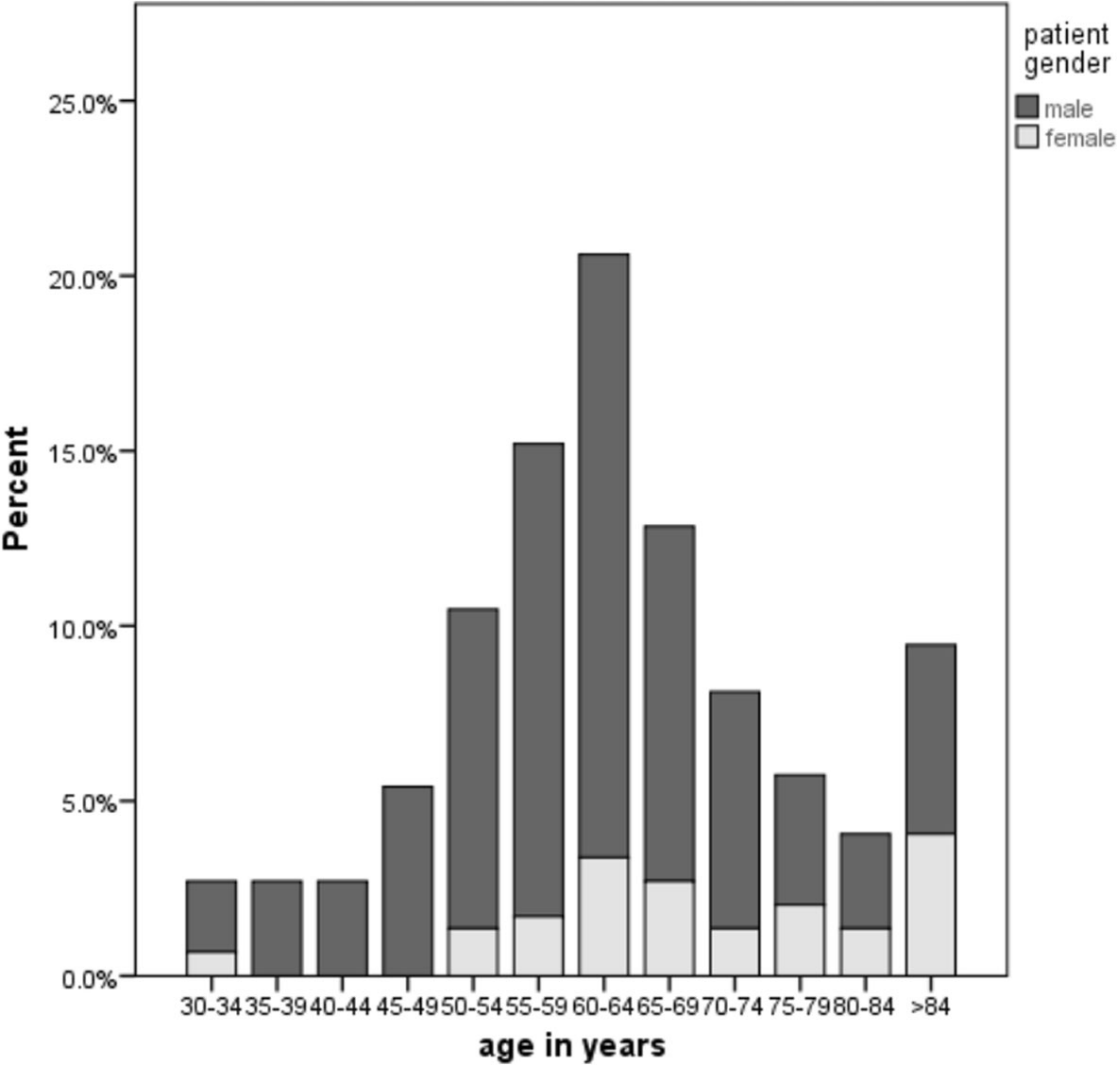
Patient age divided into 5-year intervals, split by gender, *n* = 296 AMI-patients

A total of 296 patients participated in the MEDEA FAR-EAST Study and completed the bedside interview. Among them, 241 (81.4%) were male and 55 (18.6%) female (see Table 3). The mean age of the study population was 62.9 years (median age: 63.0 years, IQR: 14 years, SD: 13. 2 years) following a near to normal distribution (see Fig. 2). The mean age of men was 61.3 years and differed significantly from women’s mean age of 69.9 years (*p* < 0.001). The mean age, the proportion of male patients and prehospital delay did not vary significantly between the four recruitment hospitals (*p* = 0.17; *p* = 0.42, *p* = 0.88). Among the study population, 29 (9.8%) had attended six years of elementary school and 114 (38.5%) an additional three years of junior-high school. 93 (31.4%) patients attained a senior-high school degree and 46 (15.5%) had a university degree.

**Table 3:** Sociodemographic, clinical and psychological characteristics of Chinese AMI-patients

	Percentage (n)	total (n)
Baseline patient characteristics		
Sex		
Male	81.4% (241)	296
Female	18.6% (55)	
Age		
Older age (≥60 years)	60.8% (180)	296
Younger age (< 60 years)	39.2% (116)	
Years of education		
6 years (elementary school)	9.8% (29)	296
9 years (middle school)	38.5% (114)	
12 years (senior high-school)	31.4% (93)	
Over 12 years (university degree)	15.5% (46)	
Less than 6 years or none	4.7% (14)	
Medical insurance		
Insured	80.5% (236)	293
Not-insured	19.5% (57)	
Clinical patient characteristics		
AMI-history		
First infarction	91.9% (272)	296
Reinfarction	8.1% (24)	
Prodromal chest pain		
Chest pain	60.1% (178)	296
No chest pain	39.9% (118)	
Psychological patients characteristics		
Anxiety (Score ≥ 10)	11.7% (31)	265
Depression (Score > 25)	12.4% (33)	267
Cardiac denial (Score ≥ 25)	53.2% (141)	265

A total of 14 patients (4.7%) had completed no education at all. 236 (80.5%) out of 293 patients were medically insured. A total of 24 (8.1%) patients had previously experienced AMI. A total of 178 (60.1%) patients experienced prodromal chest pain (PCP) prior to AMI, which could be classified as angina pectoris in 76 (25.7%) cases. Among our patient sample, 31 (11.7%) patients suffered from anxiety, 33 (12.4%) from depression, and 141 (53.2%) from cardiac denial during the 6 months prior to AMI. The response rate for all three questionnaires was at least 89.5%.

### Factors surrounding symptom-onset

Upon symptom-onset of AMI, 121 (40.9%) of patients experienced a ‘classical’ onset with chest pain and radiating pain into left arm, right arm, neck/jaw, shoulders or epigastric region (see Table 4). Chest pain without radiation was experienced by 138 (46.6%) patients. 37 (12.5%) patients stated that they did not experience any form of chest pain. 166 (61.0%) patients attributed their symptoms to cardiac origin. 200 (69.7%) patients felt that their symptom didn’t match their previous expectation of a heart attack. A majority of the patients (176 of 294, 59.9%) were driven to the hospital by either private or public transportation (see Table 4). 67 (22.8%) patients reached the hospital via ambulance. The remaining 51 (17.3%) patients stated that they walked or drove to the hospital themselves.

**Table 4:** Factors surrounding symptom-onset in Chinese AMI-patients

	Percentage (n)	total (n)
Onset of acute myocardial infarction		
In the acute situation		
Chest pain and radiation	40.9% (121)	296
Only chest pain	46.6% (138)	
No chest pain	12.5% (37)	
Symptom appraisal at symptom-onset		
Symptom attribution		
Cardiac (> 3)	61.0% (166)	272
Non-cardiac (≤3)	39.0% (106)	
Symptom expectation		
As expected (> 3)	30.3% (87)	287
Not as expected (≤3)	69.7% (200)	
Context variables of symptom-onset		
Transport		
Self-transported (walking/ driving)	17.3% (51)	294
Driven by others (private/ public)	59.9% (176)	
Via ambulance	22.8% (67)	

### Prehospital delay

**Fig. 3:**
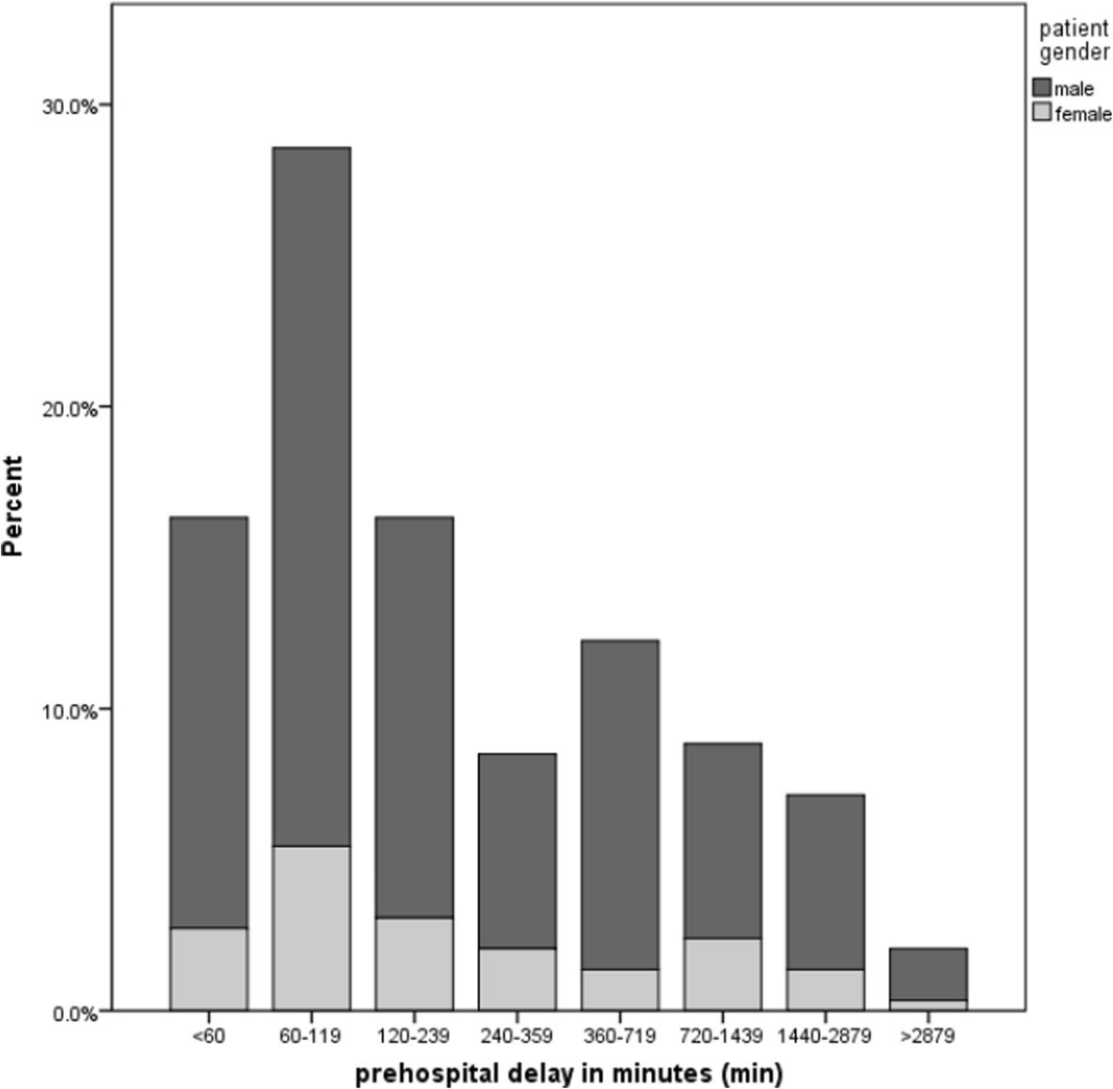
Prehospital delay (PHD) in intervals, split by gender, *n* = 294 AMI-patients

Prehospital delay was available for 294 patients. The overall median prehospital delay time in the patient sample was 150.5 min, with 152 min (IQR: 70, 495) for men and 143 min for woman (IQR: 80,662). This difference was not significant (*p* = 0.88) (see Fig. 3). A total of 132 (44.9%) patients arrived at the hospital door within a favorable time window of less than 120 min (see Fig. 3). 48 (16.3%) patients delayed within a critical time window of 120 to 239 min. In 25 (8.5%) patients, delay times between 240 and 359 min were documented. A total of 89 (30.3%) patients exhibited a detrimental delay of 360 min (6 h) and more.

## Discussion

Despite an often imperative and severe pain pattern in the face of an acute myocardial infarction, and despite increasing knowledge in the population of the need to access therapeutic help as quick as possible, inadequate delay times of AMI patients remain an universal problem with a reported average median delay time of about 204 min among 23 studies worldwide [6]. In contrast to this finding, the MEDEA FAR EAST Study including 296 AMI-patients revealed a clinically-relevant median prehospital delay of 151 min, with more than half of the patients delaying over 2 h. This finding is in line with previous investigations on median delay times in Chinese AMI-patients with a substantially lower time window ranging between 130 and 150 min over time [10, 12, 13, 15, 68].

The study has been performed in Shanghai - one of the world’s biggest cities. Nevertheless, our descriptive analysis demonstrates that the population under investigation in the MEDEA FAR-EAST Study has comparable characteristics to related investigations in China. This is particularly true concerning mean age [10, 12, 14, 16], gender distribution [12, 14], education [10] and insurance rate [12, 16].

The MEDEA FAR-EAST Study is, to the best of our knowledge, the first clinical study in the context of cardiac emergency facilities in China to comprehensively assess clinical, psychological and health-behavior related features in a homogeneous population of acute cardiac patients. In order to gain a deeper understanding of patients’ decision processes, it is also one of the first studies worldwide to assess depression, anxiety and self-perceived stress, but also the patient’s level of resilience and attitude towards the medical system. It not only screens for typical but also atypical symptoms and complaints, and furthermore assesses factors like symptom expectation and knowledge.

### Patient characteristics

Our results showed that the majority of patients were male, 60 years of age or older, and had attended school for at least six years. A majority of patients were experiencing acute myocardial infarction for the first time. About 12% of them suffered from anxiety and/or depression while cardiac denial was more common, with approximately half of the patients exhibiting cardiac denial in the prodromal phase. To date, the prevalence of depression, anxiety and cardiac denial prior to AMI has not been investigated in China. In the German counterpart study, prevalence of anxiety and cardiac denial among STEMI-patients was 11% and 42%, which correlates well with our figures [26, 29]. In a study examining depression among elderly Chinese in Malaysia, the prevalence of depression using the MDI was found to be 10.7% among 150 participants [47]. A prevalence of 12.4% in our patient sample corresponds well to that figure. The validation study of the GAD-7 scale in 600 Chinese outpatient patients found a prevalence of 4.7%. Our figure was higher, possibly explained by a higher perceived risk of MI among cardiovascular patients which has been shown to be associated with anxiety [29].

### Factors surrounding symptom-onset

In the acute situation, the vast majority of patients experienced chest pain which corresponds to figures previously reported in China [10]. “It is noteworthy that, in the current investigation, a total of 39% of patients attributed their symptoms to non-cardiac origin and around 70% of patients stated that they experienced symptoms other than they had expected. In previous studies in China, the prevalence of patients attributing their symptoms to non-cardiac origin was around 42-45%, [12, 14] which corresponds with our data, while symptom-mismatch among Chinese AMI-patients was reported to be much lower in the literature, with a figure around 31% [12]”. As a response to these symptoms most patients were driven to the hospital or walked/drove themselves. 22.6% of patients used the ambulance. A slightly higher proportion of around 31% was reported in a prehospital delay study set in Shanghai [13], which might be explained by the higher insurance rate reported in that study. The consistency of our results with Chinese literature can be seen as a measure of the validity of the data collected on AMI-patients in this investigation.

### Strengths and limitations

The strength of this study is that efforts were made to reduce recall bias by conducting a bedside interview within a narrow time-frame upon hospitalization, while additionally using an approved technique for triangulation of onset-time. Yet recall bias might still exist, as data collection was retrospective. Due to the cross-sectional study design, causal attributions cannot be made. Furthermore, despite our best efforts, consecutive inclusion is likely but cannot be fully guaranteed. The study was conducted in an urban setting in China which may prevent conclusions for rural areas and countries other than China. Furthermore, prehospital delay puts patients at risk of sudden cardiac arrest, the major cause of out-of-hospital death in the prehospital phase [69]. These patients were not included in our investigation. Furthermore, four instruments (MDI-scale, IHS-scale, RS-5-scale and CDI-scale) were not available in Chinese and had to be translated and pre-tested.

## Conclusions

Substantial progress has been made in minimizing transportation and door-to-needle time. However, patient delay remains a major unresolved concern of public health. In order to develop effective prevention campaigns for the future, the patient-related barriers to seek medical help must be better understood. Clarifying psychological and bio-behavioral factors as well as intercultural comparisons will provide key evidence for developing new approaches in prevention.

Symptom perception and miscellaneous psychological and behavioral factors may contribute equally, in a complex interaction, to inner barriers of the patient’s decision-making process. The present investigation is the first study in a Chinese population to capture comprehensive data applying theory-guided standardized instruments, allowing focus on the particularly vulnerable decision process. The prevalence of potential contributors to prehospital delay, such as symptom interpretation, and psychological barriers such as depression and cardiac denial were measured. In particular, symptom mismatch and cardiac denial were present in more than half of the patients, and we aim to conduct further analysis in the future to examine their association with prehospital delay.

Furthermore, barriers in treatment seeking behavior have been shown to vary by region and culture [70]. It is of note that this investigation strictly follows the protocol previously applied in the German MEDEA Study, enabling us, in the future, to conduct in-depth analyses of possible psychological and cultural differences in behavior and perception of threat cues during a critical and highly stressful event. Of note, in the German MEDEA-study, overall median delay time of all 619 study participants was 203 min and was 151 min in the MEDEA FAR-EAST Study - a first difference to be further clarified in future comparisons of delay between German and Chinese AMI-patients.

## Abbreviations

AMI: Acute myocardial infarction
CCU: Coronary Care Unit
CDIS: Cardiac Denial of Impact Scale
CVD: Cardiovascular disease
DS-14: Type-D Personality Scale
ECG: Electrocardiogram
EMS: Emergency Medical Services
FRAS: Framingham Type A behavior score
GAD-7: Generalized Anxiety Disorder Scale with 7 items
ICD-10: International Classification of Diseases
IHS: Interheart stress scale
IQR: Interquartile Range
MDI: Major depression inventory
MHLC: Multidimensional health locus of control scale
NSTEMI: Non-ST-elevated myocardial infarction
PCP: Prodromal chest pain
RS-4: Resilience Scale with 4 items
SD: Standard deviation
SOP: Standard operating procedures
SSRS: Social Support Rating Scale
SSS-8: Somatic symptom scale with 8 items
STEMI: ST-elevated myocardial infarction
WHO: World Health Organization
WHO-5: Well-being scale by the WHO
